# Enhancing the principles of experiential marketing in ophthalmology services

**DOI:** 10.22336/rjo.2021.73

**Published:** 2021

**Authors:** Iuliana Raluca Gheorghe, Victor Lorin Purcărea, Andreea Gabriela Schmitzer, Consuela-Mădălina Gheorghe

**Affiliations:** * Department of Marketing and Medical Technology, “Carol Davila” University of Medicine and Pharmacy, Bucharest, Romania; ** “Carol Davila” University of Medicine and Pharmacy, Bucharest, Romania

**Keywords:** experiential marketing, ophthalmological services, marketing strategy, hedonic experience

## Abstract

**Introduction**.

Consumers are getting more empowered, the process of empowerment transforming them into the partners of organizations or co-producers of services. Health care services, as well as ophthalmology services, have been a sensitive topic and raised several debates that focused on the principles of Experiential Marketing and hedonic experiences.

**Aim**.

The aim of this paper was to investigate the experiences of patients in a public ophthalmology organization by using the components of Experiential Marketing: sense, feel, think, act, and relate.

**Materials and methods**.

The case study was cross-sectional, and the sampling method was non-probabilistic, following a snowball technique. The sample was made up of 100 patients of the Clinical Emergency Eye Hospital in Bucharest. The instrument for data collection was a self-administered questionnaire. The statistical analysis was conducted in SPSS version 20 and frequencies or percentages were used for describing the qualitative data.

**Findings**.

Experiential Marketing principles may successfully be applied in public ophthalmology services if a special attention is given to the Feel and Relate components.

## Introduction

Today we are facing a dynamic evolution of the consumers’ behaviors that have suffered significant changes, becoming more experiential oriented, with a specific interest in the hedonic type, which stimulates the senses and triggers different emotions [**1**]. 

Consumers have more information in their hands, getting more empowered and transforming into partners for organizations, and, in addition, even sharing valuable information so as contributing to the outcomes of an organization in a beneficial way [**2**]. In this context, marketing specialists must focus on new strategies that offer transparency, freedom, and easiness in the exchanging information process, in order to provide value, not in a utilitarian way, but rather in the shape of a hedonistic experience [**3**].

In 1999, Bernd Schmitt mentioned the concept “experiential marketing”, emphasizing the outcome of an experience being the consumer’s triggered emotions and the activated senses, not necessarily the benefits, quality, and satisfaction [**1**]. Consequently, having in mind this concept, Schmitt divided the types of experiential marketing into five categories: sense, feel, think, act, and relate. Sense is the experience that focuses on the fact that the consumers live the moment, whereas Feel is the experience earned through a process of stimulating the emotions, feelings, and the state of mind of an individual while using the service. On one hand, Think refers to the stimulation of consumer creativity from a cognitive perspective, and Act suggests the consumer’s activities, in which he is involved, having a direct physical contact with the service, with the aim of changing different behaviors and lifestyles, and on the other hand, Relate offers the possibility of establishing connections with different communities and entities, when buying or consuming the service. Relate experience ensures that consumers build their own connections with social communities during consumption or purchase of the services. Experiential marketing has an important role in Marketing because it is based on the whole (hedonic) consumer experience of a certain service. Thus, the aim of Experiential marketing is to reach both the rational and emotional states of the people, namely living a hedonic experience. 

Organizations are using Experiential Marketing because it provides high chances of building strong customer-brand connections and, therefore, building loyalty, word-of-mouth, and brand awareness.

Most of the scientific literature about Experiential Marketing concentrates on product experience [**4**], service experience [**5**], and consumption experience [**6**], but few have focused on the health care services [**7**].

Although, the concept of the health care consumer service perception has raised attention and debates in order to eliminate the lack of information they face, the conclusion reached supported the application of experiential marketing strategies, as it is a field characterized by interdisciplinarity [**8**,**9**]. More exactly, in health care services, marketing may be efficiently applied considering the content and the features of services and the consumer-provider interaction, associated with prevention, patient support and health promotion.

The aim of this paper was to emphasize and understand the experiences of patients in the Clinical Emergency Eye Hospital in Bucharest, Romania. The experiential approach had a cognitive and emotional part, and the hedonic experiences were highlighted as perceptions, feelings, and thoughts in relation with the ophthalmological service. Experiential marketing can emphasize, as a result of the experiences of the people, the aspects that can be improved in the patient-health service relationship in Ophthalmology.

## Materials and methods

The case study was cross-sectional and the sampling method was non-probabilistic, following a snowball technique. The sample was made up of 100 patients of the Clinical Emergency Eye Hospital in Bucharest.

The instrument for data collection was a self-administered questionnaire during 1st of January and 15th of June 2021, that has already been tested and validated in an ophthalmology setting in a private health care organization [**7**]. As such, the questionnaire consisted of 2 sections: the first section contained several demographic questions, and the second section encompassed closed end questions related to the perception of public ophthalmologic services from an experiential marketing perspective through its components, sense, feel, think, act, and relate, respectively. All the items of the second section in the questionnaire were measured on a 5-point Likert scale ranging from 1 with Strongly Disagree to 5 to Strongly Agree.

The statistical analysis was conducted in SPSS version 20 and frequencies or percentages were used for describing the qualitative data, being depicted in figures.

## Findings

**Fig. 1:**
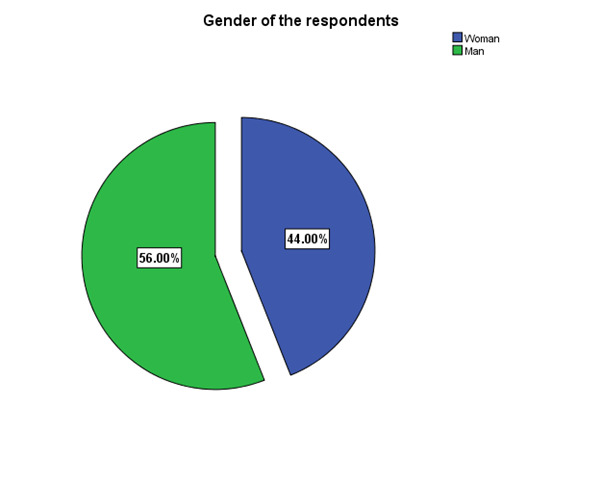
Gender of respondents

**Fig. 2:**
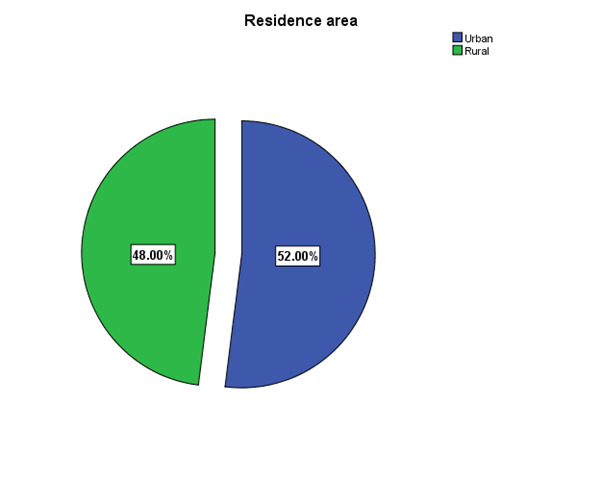
Residence area

**Fig. 3:**
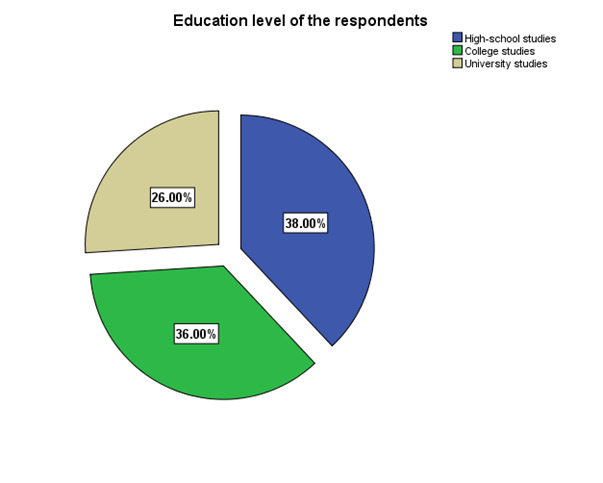
Education level of respondents

According to the demographic profile of the patients, the vast majority were males (56%), from urban areas (52%), and had high-school studies (38%) (**Fig. 1**-**3**).

**Fig. 4:**
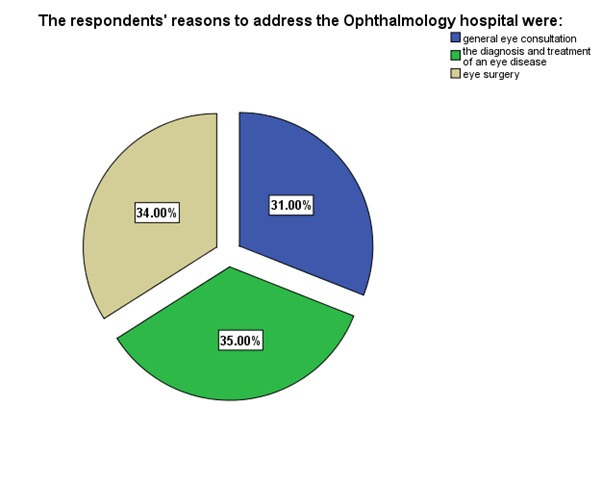
The respondents’ reasons to address the Ophthalmology hospital

**Fig. 4** shows the respondents’ reasons to address the Ophthalmology hospital: general eye consultation (31%), diagnosis and treatment of an eye disease (35%) and eye surgery (34%).

**Fig. 5:**
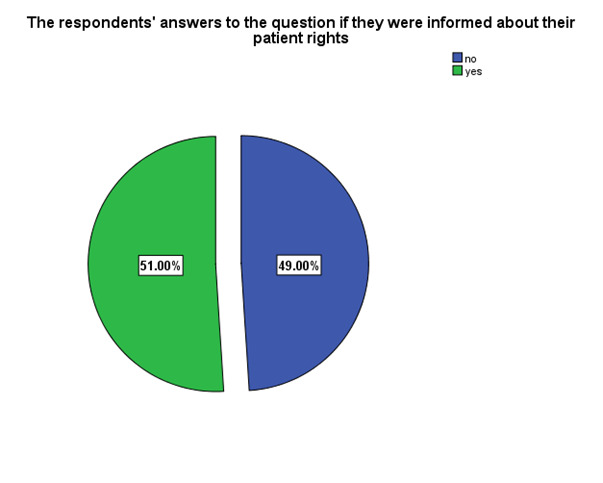
Patients’ perception of being informed of their rights

According to **Fig. 5**, 51% of the respondents confirmed that they were informed about their patient rights and 49% considered that they were not informed. 

**Fig. 6:**
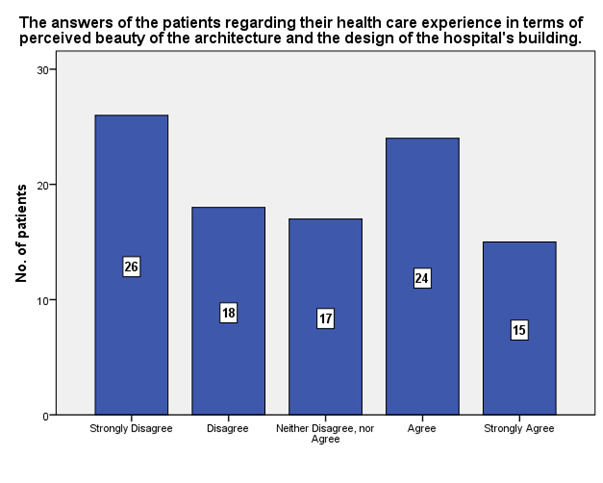
Opinions of the patients regarding the experience in terms of perceived beauty of the architecture and the design of the hospital’s building

**Fig. 6** shows that most of the patients (26) considered that the architecture and the design of the whole building of the ophthalmology hospital were not beautiful, while 24 considered the opposite.

**Fig. 7:**
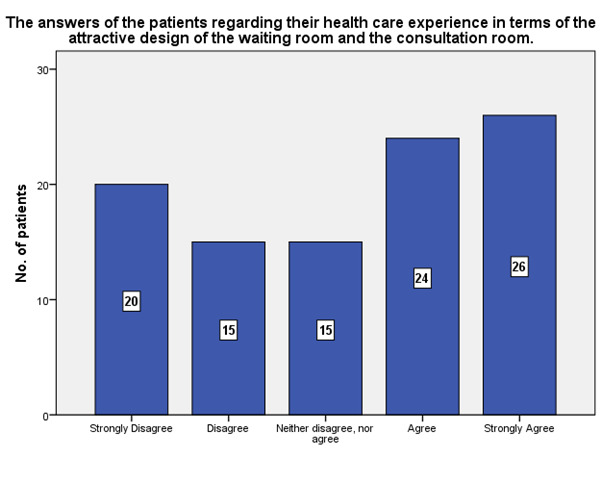
Patients’ experience in terms of the attractive design of the waiting room and the consultation room

According to **Fig. 7**, the design of the waiting room and the consultation room were considered attractive by 26 patients, while 20 patients considered the opposite.

**Fig. 8:**
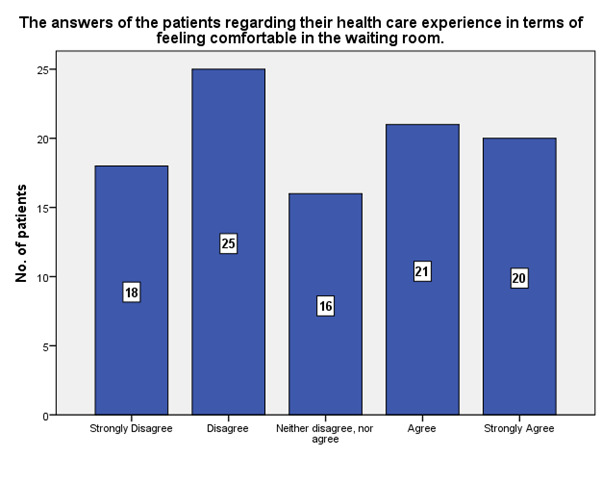
The waiting room made the patients feel comfortable

**Fig. 8** shows to which extent the waiting room made the patients feel comfortable, 25 of them declared that it did not make them feel comfortable, while 20 declared the opposite.

**Fig. 9:**
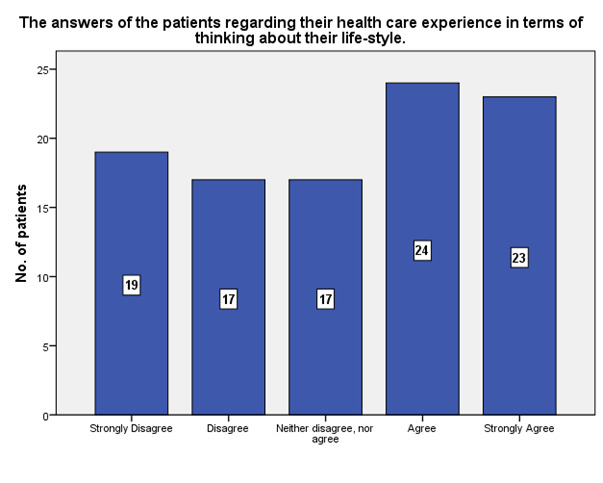
The experience in the ophthalmology hospital and the lifestyle

According to the patients, 24 agreed that the experience in the ophthalmology hospital made them think about their lifestyle, while 19 strongly disagreed (**Fig. 9**). 

**Fig. 10:**
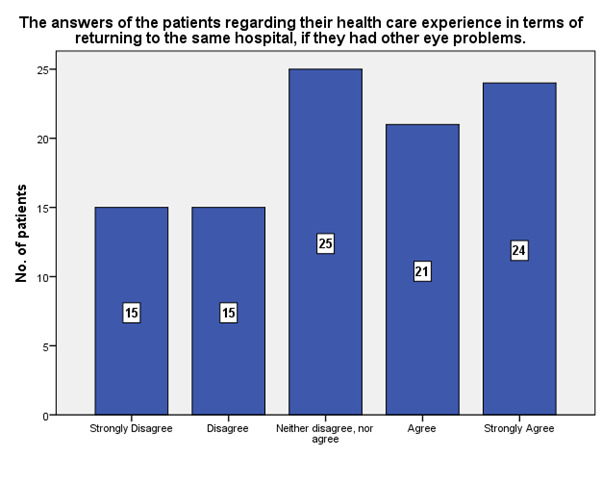
Experience of the patients and returning to the same ophthalmology hospital in case of other eye problems

According to the study results, 24 patients declared that if they ever had other eye problems, they would return to the same medical hospital (**Fig. 10**). 

**Fig. 11:**
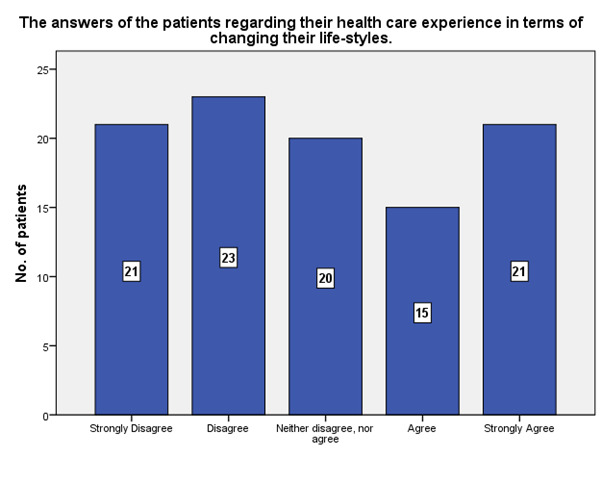
The experience in terms of changing the patients’ lifestyles

According to the patients, 21 strongly agreed that the experience in the ophthalmology hospital made them change their lifestyle, while 21 strongly disagreed (**Fig. 11**).

**Fig. 12:**
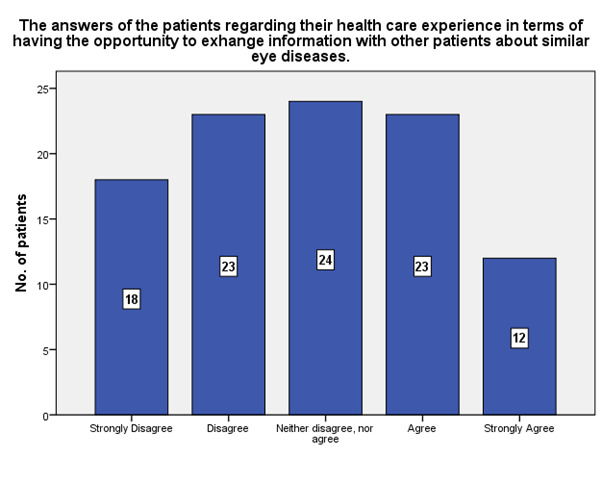
The experience of the patients regarding the possibility to exchange information with other patients about similar eye disease

According to **Fig. 12**, only 12 strongly agreed that going to the ophthalmology hospital offered the possibility to exchange information with other patients about similar eye diseases and 18 strongly disagreed.

**Fig. 13:**
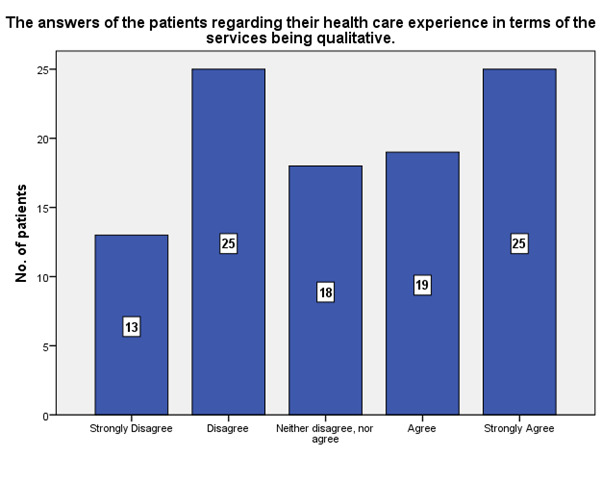
The experience of the patients regarding the quality of the ophthalmological health service received

According to the patients’ experiences, 25 strongly agreed that the ophthalmological health service received was qualitative, while 13 strongly disagreed (**Fig. 13**).

**Fig. 14:**
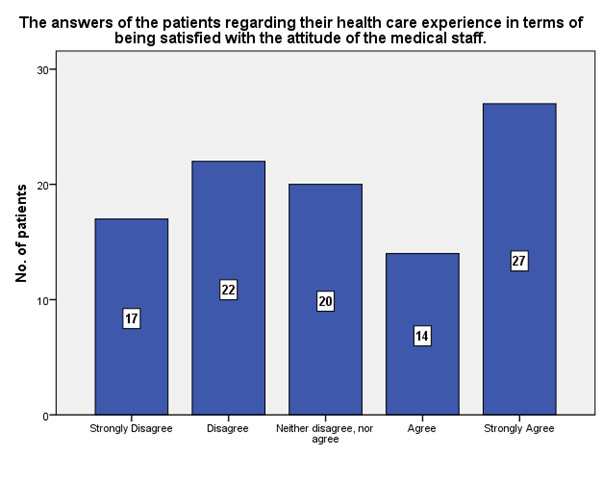
The experience of the patients regarding their satisfaction with the attitude of the medical staff

According to the patients’ experience, 27 strongly agreed that they were satisfied with the attitude of the medical staff, while 17 strongly disagreed (**Fig. 14**).

**Fig. 15:**
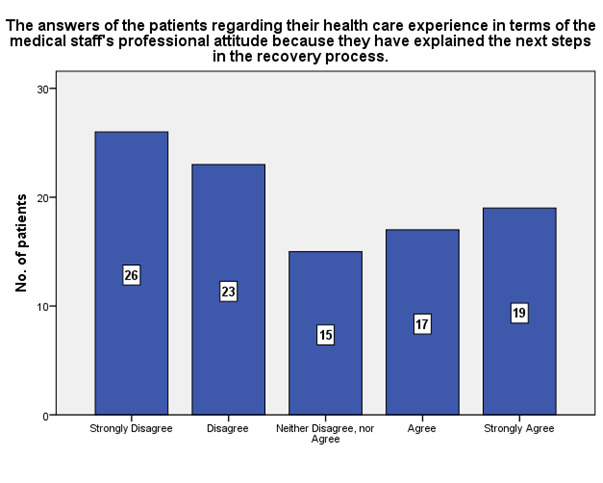
The answers of the patients regarding their health care experience in terms of the medical staff’s professional attitude because they have explained the next steps in the recovery process

According to the patients’ experience, 19 of them strongly agreed that the medical staff had a professional attitude because they have explained the next steps in the recovery process, while 26 strongly disagreed (**Fig. 15**).

**Fig. 16:**
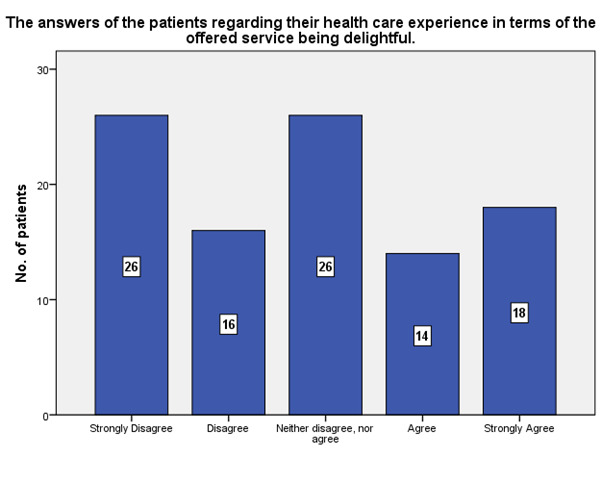
The experience of the patients regarding their health care experience in terms of the offered service

According to the patients’ experience, 18 of them strongly agreed that the service offered was delightful, while 26 strongly disagreed (**Fig. 16**).

**Fig. 17:**
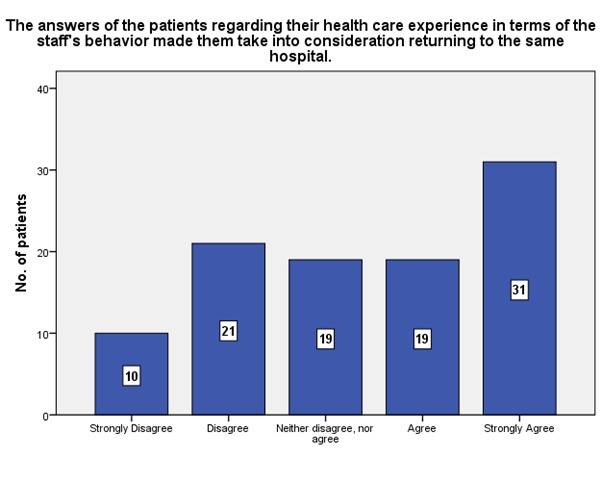
The experience of the patients in terms of the staff’s behavior that made them take into consideration returning to the same hospital

According to the patients’ experience, 31 of them strongly agreed that the staff’s behavior made them take into consideration returning to the same hospital (**Fig. 17**).

**Fig. 18:**
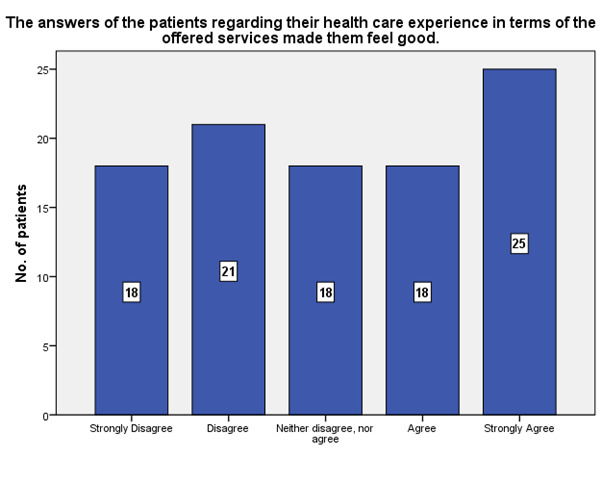
The experience of the patients in terms of the offered services that made them feel good

According to the patients’ experience, 25 of them strongly agreed that the offered services received made them feel good, while 18 strongly disagreed (**Fig. 18**).

**Fig. 19:**
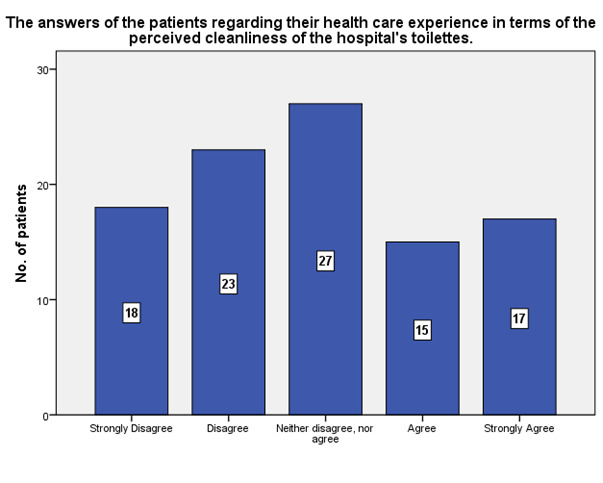
The experience of the patients in terms of the perceived cleanliness of the hospital’s toilettes

According to the patients’ experience, 17 of them strongly agreed that the hospital’s toilettes were perceived as clean, while 18 strongly disagreed (**Fig. 19**).

**Fig. 20:**
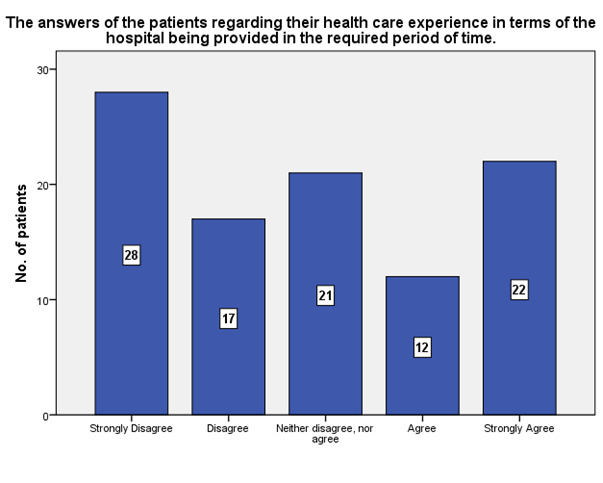
The experience of the patients regarding the offering of the services in the required period of time

According to the patients’ experience, 22 of them strongly agreed that the answers of the patients regarding their health care experience in terms services of hospital being provided in the required period of time, while 28 strongly disagreed (**Fig. 20**).

**Fig. 21:**
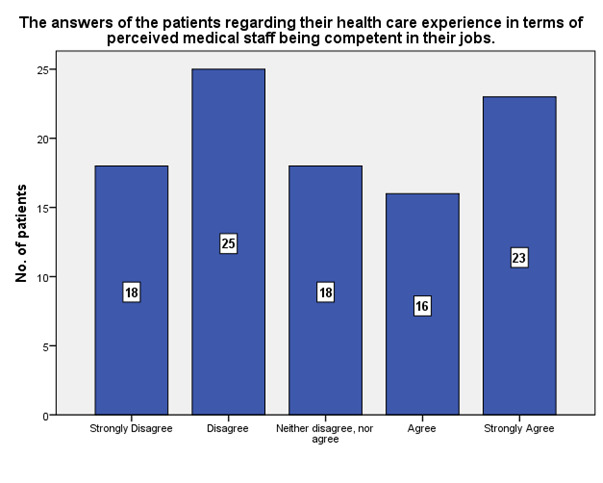
The experience of the patients regarding the competence of the medical staff

According to the patients’ experience, 23 of them strongly agreed that the medical staff was competent in their jobs, while 18 strongly disagreed (**Fig. 21**).

**Fig. 22:**
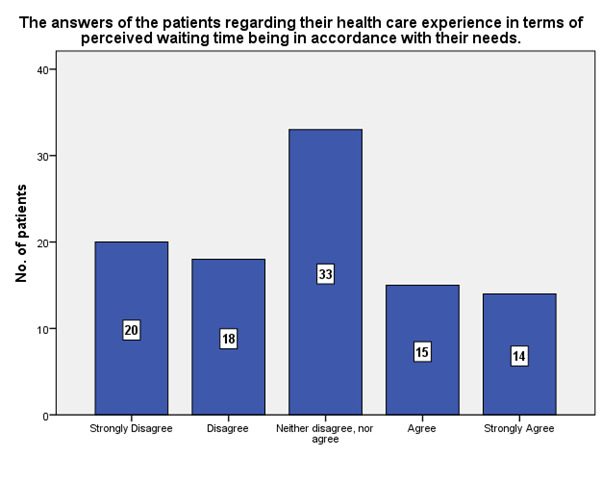
The experience of the patients regarding the perceived waiting time in accordance with their needs

According to the patients’ experience, 14 of them strongly agreed that the waiting time was in accordance to their needs, while 20 strongly disagreed (**Fig. 22**).

**Fig. 23:**
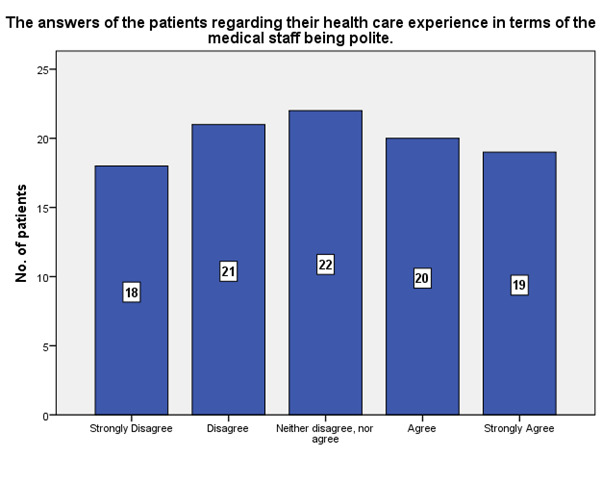
The experience of the patients regarding the politeness of the medical staff

According to the patients’ experience, 19 of them strongly agreed that the medical staff was polite, while 18 strongly disagreed (**Fig. 23**).

**Fig. 24:**
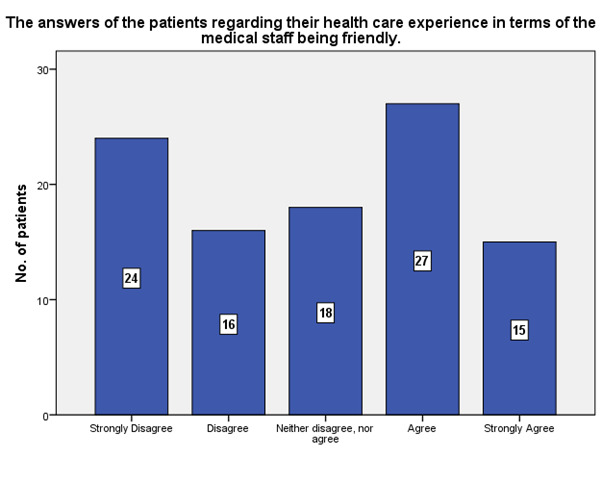
The experience of the patients regarding the friendliness of the medical staff

According to the patients’ experience, 15 of them strongly agreed that the medical staff was friendly, while 24 strongly disagreed (**Fig. 24**).

**Fig. 25:**
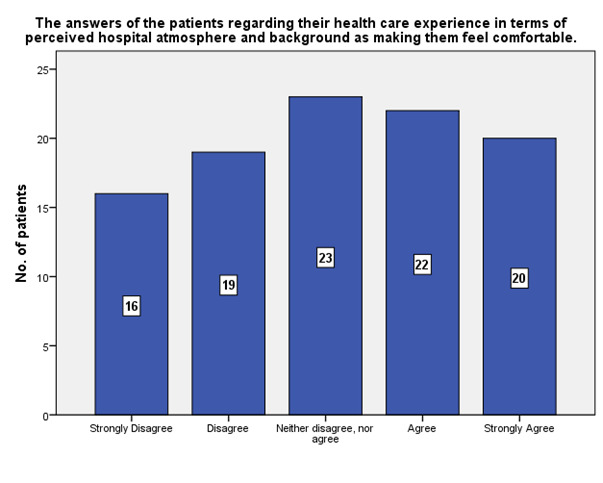
The experience of the patients in terms of perceived hospital atmosphere and background

According to the patients’ experience, 20 of them strongly agreed that the hospital atmosphere and background made them feel comfortable (**Fig. 25**).

**Fig. 26:**
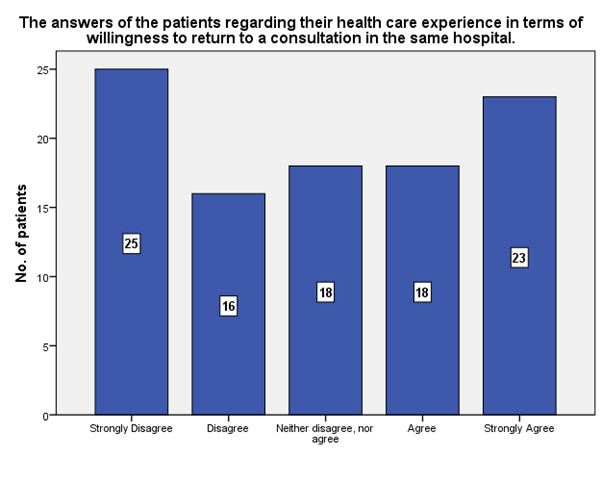
The answers of the patients regarding their health care experience in terms of willingness to return to a consultation in the same hospital

According to the patients’ answers, 23 of them strongly agreed that they will return to a consultation in the same hospital, while 25 strongly disagreed (**Fig. 26**).

**Fig. 27:**
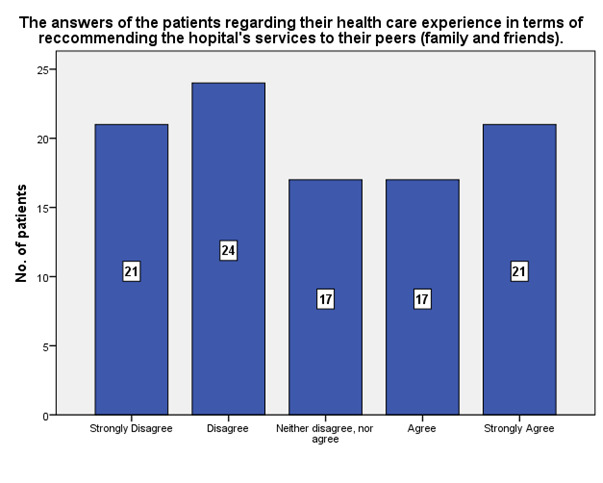
The answers of the patients regarding their health care experience in terms of recommending the hospital’s services to their peers (family and friends)

According to the patients’ answers, 21 of them strongly agreed that they will recommend the hospital’s services to their peers (family and friends), while 21 strongly disagreed (**Fig. 27**).

## Discussion

Nowadays, regardless of the field, specialists use experience to influence a target audience, because it was concluded that there is always an effective outcome, because of using an experience in distributing a message or content (a behavioral response) [**10**]. 

However, there are fields, such as health care, that are sensitive to be addressed even through experiential marketing strategies. As our research findings suggested, the experiential marketing components that require attention in Ophthalmology public services are Feel and Relate, this result being in accordance with previous literature [**11**]. 

Experiential marketing is a valuable strategy that ophthalmologic organizations may use to target specific consumers, through a compelling service experience in a hedonic manner. Building personally relevant and memorable experiences will make consumers feel comfortable with their choices and will relate more easily with other peers in an online or offline environments [**12**].

The core principles of Experiential Marketing, that may also be applied in Ophthalmology services, are connected to communication by using touch and emotions in helping consumers grasp the health care service, and promote the use and benefits of consuming the service in an aspirational manner, by delivering, in fact value in a hedonic way [**13**].

## Conclusions

Experiential Marketing principles may successfully be applied in public ophthalmology services if a special attention is given to the Feel and Relate components. The key to make consumers perceive the principles of Experiential Marketing in a positive manner is to offer them hedonic experiences. 

**Conflict of Interest statement**

Authors state no conflict of interest.

**Informed Consent and Human and Animal Rights statement**

Informed consent has been obtained from all individuals included in this study.

**Acknowledgements**

None.

**Sources of Funding**

None.

**Disclosures**

None.
